# Expression analysis of the CLCA gene family in mouse and human with emphasis on the nervous system

**DOI:** 10.1186/1471-213X-9-10

**Published:** 2009-02-11

**Authors:** Marko Piirsoo, Dies Meijer, Tõnis Timmusk

**Affiliations:** 1 Department of Gene Technology, Tallinn University of Technology, Akadeemia tee 15, Tallinn 19086, Estonia; 2 Department of Cell Biology and Genetics, Erasmus University Medical Center, PO box 2040, 3000CA Rotterdam, the Netherlands

## Abstract

**Background:**

Members of the calcium-activated chloride channel (CLCA) gene family have been suggested to possess a variety of functions including cell adhesion and tumor suppression. Expression of CLCA family members has mostly been analyzed in non-neural tissues. Here we describe the expression of mouse and human CLCA genes in the nervous system.

**Results:**

We show that from the six mouse CLCA family members only Clca1, Clca2 and Clca4 mRNAs are expressed in the adult brain, predominantly in olfactory ensheathing cells. During mouse nervous system development Clca1/2 is more widely expressed, particularly in cranial nerves, the diencephalon and in the cerebral cortex. While human CLCA2 and CLCA4 genes are widely expressed in brain, and at particularly high levels in the optic nerve, human CLCA3, the closest homologue of mouse Clca1, Clca2 and Clca4, is not expressed in the brain. Furthermore, we characterize the expression pattern of mouse Clca1/2 genes during embryonic development by in situ hybridization.

**Conclusion:**

The data published in this article indicate that within the nervous system mouse Clca1/2 genes are highly expressed in the cells ensheathing cranial nerves. Human CLCA2 and CLCA4 mRNAs are expressed at high level in optic nerve. High level expression of CLCA family members in mouse and human glial cells ensheathing nerves suggests a specific role for CLCA proteins in the development and homeostasis of these cells.

## Background

Calcium activated chloride currents have been characterized in a number of cell types including smooth muscle, skeletal muscle and epithelium. Physiologically, it has been shown that activation of calcium-activated chloride current plays a prominent role in among others the maintenance of smooth muscle tone, epithelial secretion and vertebrate olfactory transduction. The precise molecular identities of the currents are still hotly debated. Proteins belonging to CLC, CLCA, bestrophin and tweety gene families have been proposed to function as calcium activated chloride channels [reviewed in [1]].

The CLCA gene family includes 4 genes in humans, 5 genes in rat and 6 genes in mouse. The nomenclature of the CLCA genes in different organisms is somewhat confusing since the numbering of different genes does not reflect the actual homologies between the genes in different organisms but rather the time of characterization [reviewed in [2]].

Although a lot of evidence shows the involvement of CLCA proteins in mediating chloride conductance, it is still unclear whether CLCA proteins are channels themselves. It has been shown that there are differences in endogenous chloride current characteristics in normal versus CLCA over-expressed cells [3]. Also, at least some of the CLCA family members appear to be secreted proteins [4,5]. Recently, using protein structure prediction, it has been proposed that CLCA proteins are membrane anchored or secreted metal-dependent hydrolases [6].

In addition to their functions as chloride channels or channel modulators, some CLCA family members function as cell adhesion molecules [7,8] and tumor suppressor proteins [9]. It has also been proposed that CLCA family members are involved in respiratory diseases like asthma [10,11] and cystic fibrosis [12].

Expression analysis by RT-PCR of mouse Clca family members, has revealed that mClca1 is expressed at high levels in spleen and bone marrow and mClca2 in mammary gland. Moderate or low expression levels of both genes were found in most tissues with only mClca1 expressed in brain tissue [13]. Expression analysis of mClca3, a secreted member of the CLCA family, has been performed by immunohistochemistry. The mClca3 protein was found only in the mucine granule membranes of the gastrointestinal and respiratory tract, uterine goblet cells and other mucin producing cells [14]. RT-PCR analysis revealed that mClca4 mRNA is also expressed in the gastrointestinal tract, as well as uterus, skeletal muscle, heart and lung [15]. RT-PCR analysis of mClca5 and 6 showed high expression of mClca5 in mouse eye and spleen, whereas mClca6 is expressed highly in the gastrointestinal tract [16].

To date, expression analysis has shown that only mClca1 is expressed in the mouse brain. However, in case of mClca3 and 4, brain tissue was not included in the expression analysis. In this study we describe the spatio-temporal expression of mClca1, 2 and 4 genes in the nervous system. We show that these genes are expressed in the olfactory ensheathing cells. In addition we also describe the expression pattern of human CLCA2 and 4 genes in the nervous system. Finally, we describe the expression pattern of mClca1/2 during embryonic development by in situ hybridization.

## Methods

### RT-PCR analysis

Total RNA from NMRI mouse tissues and total RNA from postmortem adult human brain regions was extracted using RNAWiz (Ambion) according to the manufacturers instructions. Total RNA from human non-neural tissues was obtained from Clontech. First-strand cDNAs were synthesized with Superscript III (Invitrogen) reverse transcriptase using 5 μg RNA as recommended by the manufacturer.

PCR reactions were performed in a volume of 25 μl, using 1/50 of the first-strand cDNA reaction. Annealing temperature for different sets of primers ranged from 55–60°C. The number of cycles used varied from 25–35 for different primer sets. Number of cycles for different primer sets was determined empirically and we always analysed the PCR product in the exponential phase of amplification. PCR with primers specific for housekeeping gene HPRT and GAPDH were used as a control to determine the variation of the amount of cDNA in different PCR reactions.

Real-time quantitative (Q) RT-PCR analysis of CLCA mRNA levels in adult mouse and human brain regions and during mouse brain development was performed in triplicates using qPCR Core Kit for SYBR Green I (Eurogentec) with Lightcycler 2.0 (Roche) according to manufacturers instructions. Data was normalized with housekeeping gene HPRT and analyzed with Lightcycler 4.05 software (Roche). Data was not normalized with HPRT in case of mClca1, mClca2 and mClca4 PCRs using equal amounts of cDNAs from different mouse brain developmental stages, since the level of HPRT mRNA is increased during development (Piirsoo and Timmusk, unpublished data).

Primers used in the experiments are the following:

hclca1 sense ACGAACAAGGACACCAGCAAA

hclca1 antisense AAGAGATCAGGTATGGGAGCAT

hclca2 sense TGCATGTCAATCACTCTCCCA

hclca2 antisense GAGTTCCTATCCATTGCTCGT

hclca3 sense GAAGGAGCTCAAACAGACGAC

hclca3 antisense ACTTTCTACTGAACCAGGCTC

hclca4 sense GCCACAGTTCATGAGGATAAG

hclca4 antisense CACAGACAATACCAGCGTAG

mclca1sense CACCAGGATCACTGGCACCAAT

mclca1 antisense GCATCGATAAGGCTGTTTAGGTC

mclca2 sense CGCCAGCATCACAGGCAAGAAG

mclca2 antisense GCGTCGATAAGGCTGCTTACATG

mclca4 sense TTCAGCAGGACAGCATCTGG

mclca4 antisense TGCCACTTGTGCGATGTTG

gapdh sense TTCCTACCCCCAATGTGTCCGTC

gapdh antisense ACCCTGTTGCTGTAGCCGTATTCA

hprt sense GATGATGAACCAGGTTATGAC

hprt antisense GTCCTTTTCACCAGCAAGCTTG

hclca2real sense AGCACCTGGAGAAGACTTTGA

hclca2real asense CTTGCTGAGGATTTCGCTTTGA

hclca4real sense AGACCTTGATGCCACAGTTCAT

hclca4real asense TGGTGACAGATCAGTAGTATTTA

mclca1 realS CACTGATAACTTGCGTATCTAC

mclca1 realAS CACAGTTGTGAACCACATTGG

mclca2 realS TCACTGATAACTTGCGTATCTAT

mclca2 realAS ACACTCGTGGACCACCTTCT

mclca4 realS AATGACAGCTCCTACCTAGC

mclca4 realAS GGCTCCACTGTGTTTGACCT

### In situ hybridization

DNA fragments for riboprobe generation were subcloned into pCRII-TOPO vector (Invitrogen), sense and antisense cRNA probes were synthesized with the MAXIScript In Vitro Transcription Kit (Ambion) T7 or SP6 RNA polymerase, using [α-^35^S]UTP (Amersham Biosciences, UK) for labeling. The hybridization specificity was confirmed using [α-^35^S]-labeled sense riboprobes synthesized from the same templates. All sense probes resulted in the hybridization signal equivalent to the background. This shows that cRNA labeling of different CLCAs was specific. Primers used to generate probes were the following:

CLCA12 sense ATAGTATCTCTGCACTGGTG

CLCA12 antisense GAATGGATATCTAATTTCCATAG

CLCA4 sense CCTCCTGGTCTGGGTACCAA

CLCA4 antisense ATAGACGCAAATAGGAAATTTAC

Serial saggital and coronal sections (14 μm) from fresh-frozen NMRI mouse brain were analyzed by in situ hybridization analysis following the previously described protocol [17]. Emulsion-dipped sections were developed after 3 weeks using D-19 developer (Eastman Kodak, USA), fixed (sodium fixer; Kodak), and counterstained with hematoxylin (Shandon, USA).

## Results

### Expression pattern of mouse Clca 1, 2 and 4 in the central nervous system

**Figure 1:**
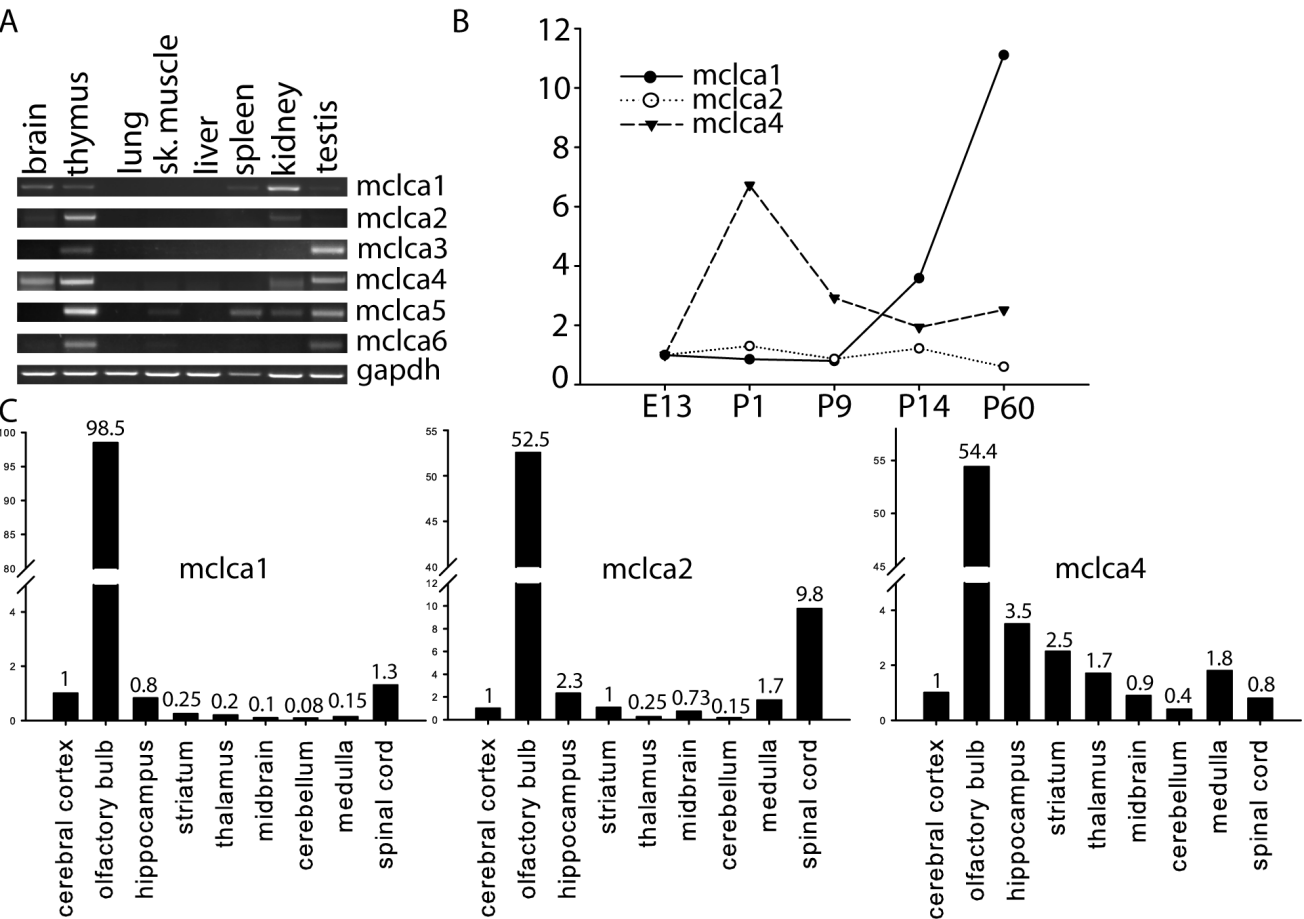
**A) Semiquantitative RT-PCR analysis of mClca1-mClca6 and control mRNA gapdh expression in mouse tissues**. B) Real-time PCR analysis of mClca1, mClca2 and mClca4 expression in the brain at indicated developmental timepoints. Expression level is shown relative to the expression level of the respective mRNA at embryonic day 13. C) Real-time PCR analysis of mClca1, mClca2 and mClca4 expression in mouse brain regions. Expression levels are shown relative to the expression level in the cerebral cortex.

Previous analyses have shown that out of six mouse Clca genes only mClca 1 is expressed in the brain and is expressed at relatively low levels compared to other tissues where the gene is expressed [1]. Our RT-PCR analysis with cDNAs from adult mouse brain showed that in addition to mClca1, mClca2 and 4 are expressed at low levels in mouse brain. In accordance with previously published data we could not detect mClca3, 5 and 6 expression in adult mouse brain (Fig 1A). All six mouse Clca genes were expressed in thymus. In addition to brain and thymus we detected mClca1 expression in spleen, kidney and testis. mClca2 was expressed in brain, thymus and kidney, mClca3 in thymus and kidney, mClca4 in brain, thymus kidney and testis. mClca5 was most widely expressed in mouse tissues, with the highest expression in thymus and lower level of expression in skeletal muscle, spleen, kidney and testis. mClca6 was expressed in thymus, skeletal muscle and testis (Fig 1A).

Quantitative real-time PCR analysis of mClca1, 2 and 4 expression during mouse brain development showed that expression of mClca1 was increasing during postnatal brain development and reached maximum levels in the adult mouse brain. mClca2 expression did not change during mouse brain development and mClca4 expression was low during embryonic development, highest around birth of the animal and the level of respective mRNA was gradually decreasing during postnatal development (Fig 1B).

In order to analyze the spatial distribution of mClca1, 2 and 4 mRNA expression in the adult brain we performed real-time PCR anaysis using cDNAs from various regions of mouse brain. Strikingly, all the mClca genes expressed in the nervous system, were highly enriched in olfactory bulb (Fig 1C). Expression levels were quantified relative to the expression in cerebral cortex. mClca1 was expressed at 98 times higher level in olfactory bulb than in cerebral cortex. mClca2 and mClca4 were expressed at 52 and 54 times higher level in olfactory bulb than in cerebral cortex. mClca2 was expressed at 10 times higher level in spinal cord than in cerebral cortex (Fig 1C).

**Figure 2:**
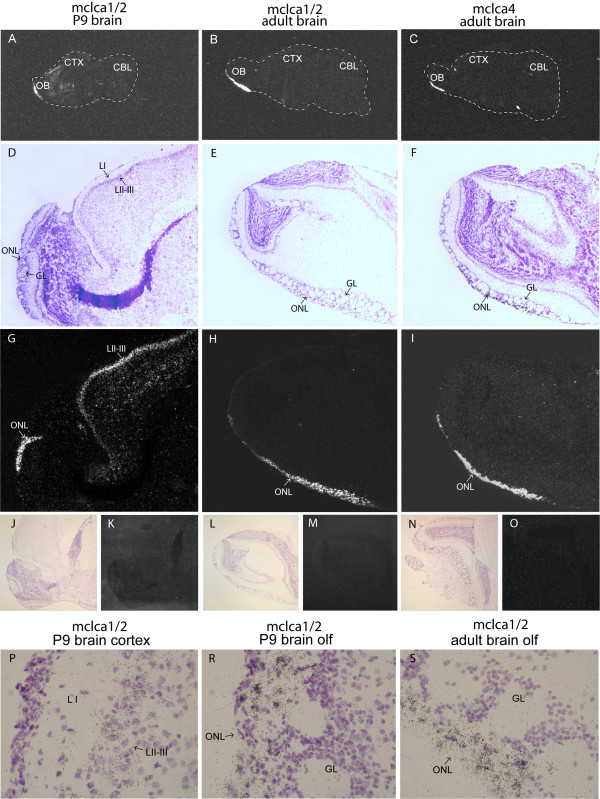
**In situ hybridization analysis of mClca1/2 and mClca4 expression in the mouse brain**. Dark-field emulsion autoradiographs of whole P9 and adult brain mid saggital sections are shown in A-C. Hematoxylin-eosin stained bright-field images (D, E and F) and corresponding dark-field emulsion autoradiographs (G, H and I) are shown at 40× magnification. Lack of in situ hybridization signal using corresponding sense probes is shown in J-O. Cellular distribution of Clca1/2 expression in P9 mouse cerebral cortex and olfactory bulb and adult mouse olfactory bulb are shown in J-L at 600× magnification. Abbreviations: CBL – cerebellum; CTX – cortex; GL – glomerular cell layer; LI – cerebral cortex layer I; LII-III – cerebral cortex layers II-III; OB – olfactory bulb; ONL – olfactory nerve layer.

To further analyze the cellular distribution of mClca1,2 and 4 expression in brain, we performed in-situ hybridization on adult brain sections. Since mClca1 and 2 genes share 95% identity, we were unable to design probes that distinguish between these genes. Therefore, we consider the expression pattern of mClca1 and 2 together (marked mClca1/2). It should be noted however, that mClca2 was expressed at very low levels in the adult mouse brain (Fig 1A) and therefore most of the signal likely corresponds to mClca1 expression. Sense probes were used as negative controls and they did not give any signal (Fig 2, J–O). Our analysis showed that mClca1/2 and 4 genes are expressed in the olfactory nerve layer of the adult brain (Fig 2, B, C, E, F, H and 2I). High magnification imaging of mClca1/2 expression in the adult mouse brain showed expression in cells next to the glomerular layer of the olfactory bulb, namely layer of entering olfactory nerve fibers, which is populated by the olfactory ensheathing cells (Fig 2, S). We observed very low levels of mClca1/2 and mClca4 expression in hypothalamic nuclei and mClca1/2 expression in the layer II-III of the cerebral cortex (data not shown). Since mClca1 seems to be the most highly expressed CLCA family member in the mouse brain, we analyzed mClca1/2 expression also in postnatal day (P9) brain. In P9 brains the expression of mClca1/2 was more broad with the highest levels in the olfactory nerve layer of the olfactory bulb (Fig 2, A, D, G, R) and also in the layer II-III of the developing cerebral cortex containing pyramidal neurons (Fig 2.A, D, G, P). At P9 low level of mClca1/2 expression was observed in CA3 layer of hippocampus and in amygdalohippocampal area (data not shown).

### Expression pattern of mouse Clca1/2 during embryonic development

**Figure 3:**
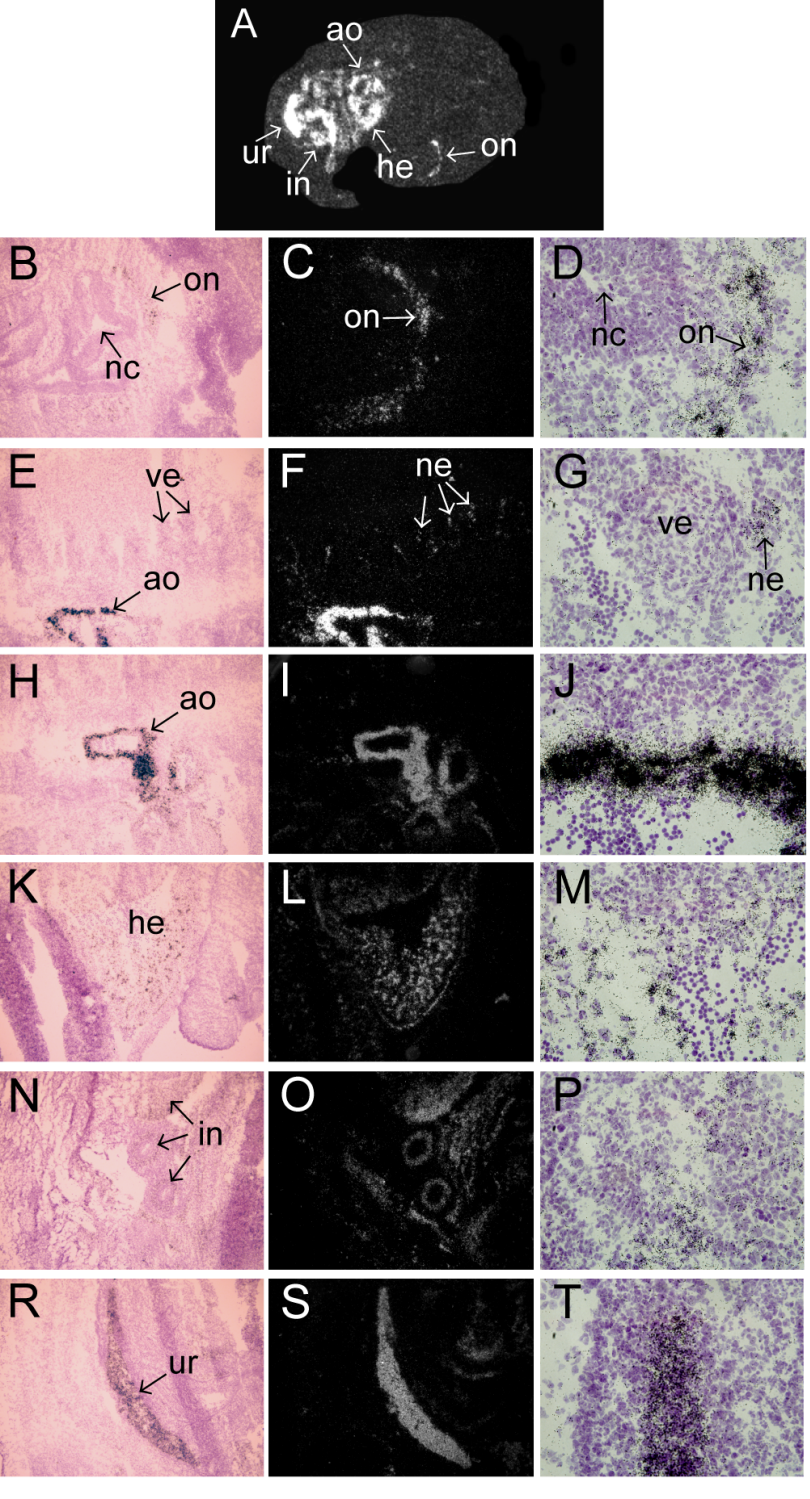
**In situ hybridization analysis of mClca1/2 expression in E13 mouse embryo**. Dark-field emulsion autoradiograph depicting whole embryo is shown in A. Hematoxylin-eosin stained bright-field images (B, E, H, K, N, R at 100× magnification and D, G, J, M, P, T at 600× magnification) and corresponding dark-field emulsion autoradiographs (C, F, I, L, O and S) are shown corresponding to various parts of the embryo. Abbreviations: ao-aorta; he-heart; in-intestine; on-olfactory nerve nc-nasal cavity; ne-spinal nerve; ur-urethra; ve-vertebrae.

We performed in situ hybridization analysis in order to characterize mClca1/2 expression during mouse development. Our analysis showed that at embryonic day 13 (E13) mClca1/2 is expressed at high levels in the developing urethra, midgut, aorta and heart (Fig 3A). High magnification analysis showed that mClca1/2 is also expressed in cells that lie adjacent to the epithelium of the nasal cavity. Most likely this expression conforms to the developing cranial nerve I (olfactory nerve)(Fig 3B–D). Low levels of mClca1/2 expression was seen in the developing spinal nerves (Fig 3E – G). In the E13 heart mClca1/2 was expressed in ventricle and atrium as well as in aorta (Fig 3H–M). High level of mClca1/2 expression was observed in E13 midgut and urethra (Fig 3N–T).

**Figure 4:**
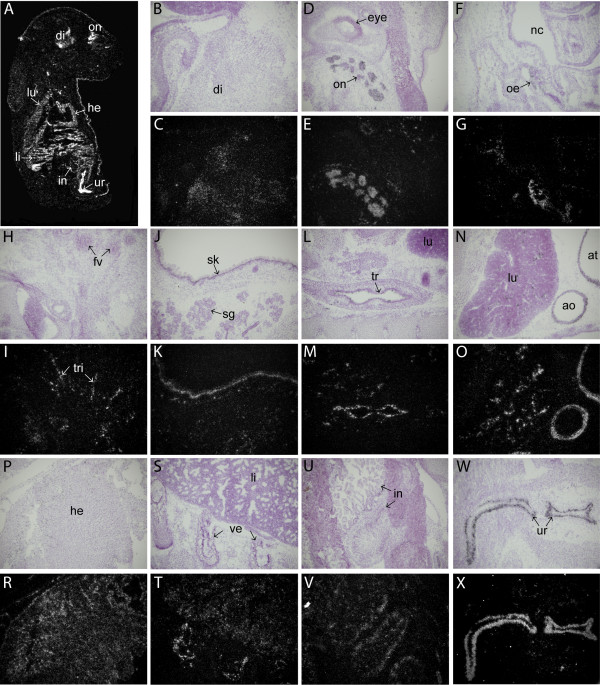
**In situ hybridization analysis of mClca1/2 expression in E17 mouse embryo**. Dark-field emulsion autoradiograph depicting whole embryo is shown in A. Hematoxylin-eosin stained bright-field images (B, D, F, H, J, L, N, P, S, U, W) and corresponding dark-field emulsion autoradiographs (C, E, G, I, K, M, O, R, T, V, X) taken at 100× magnification, corresponding to various parts of the embryo are shown. Abbreviations: di-diencephalon; fv-follicles of vibrissae; he-heart; in-intestine; li-liver; lu-lung; oe-olfactory epithelium; on-optic nerve; sg-submandibular gland; sk-skin; tr-trachea; tri-trigeminal nerve; ur-urethra; ve-vertebrae.

**Figure 5:**
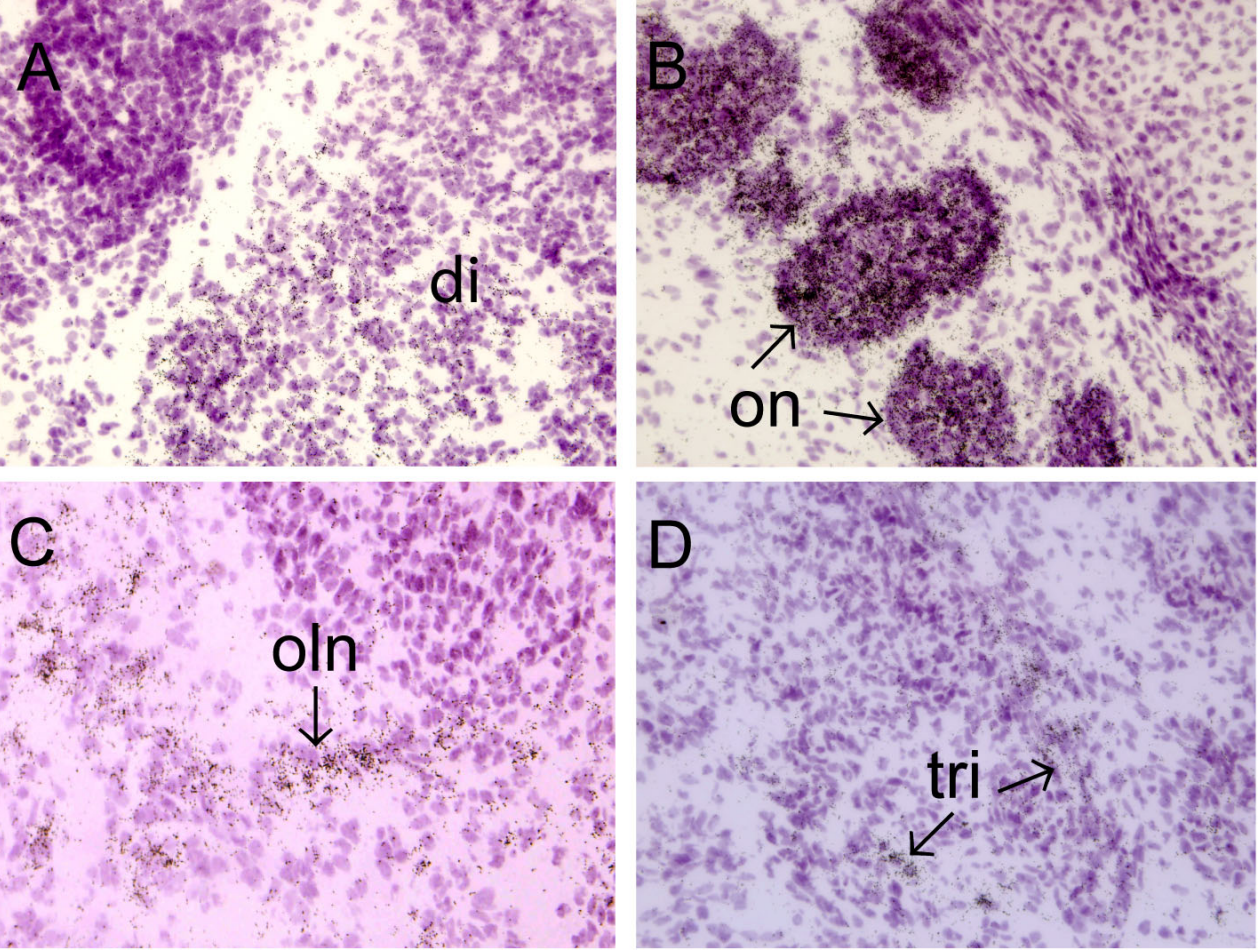
**High magnification images of mClca1/2 expression in the nervous system at E17**. Images of diencephalon (A), optic nerve (B), olfactory nerve (C) and trigeminal nerve (D) are shown at 600× magnification. Abbreviations: di-diencephalon; oln-olfactory nerve; on-optic nerve; tri-trigeminal nerve.

In situ hybridization analysis on E17 mouse embryos showed that high levels of mClca1/2 mRNA expression were retained in the developing urethra (Fig 4A). Expression was seen also in the brain, predominantly in the diencephalon (Fig 4B,C). Highest level of mClca1/2 expression in the nervous system was observed in optic nerve (Fig 4D,E). The expression of mClca1/2 was also detected in the olfactory nerve (Fig 4F,G) and in the trigeminal nerve (Fig 4H,I). Higher magnification images of mClca1/2 expression in the nervous system of E17 embryos are shown in Fig 5. Outside the nervous system the expression of mClca1/2 was observed in the heart (Fig 4N–R), intestine (Fig 4U,V), urethra (Fig 4W–X) and also in trachea and lung (Fig 4L–O). Lower levels were seen in liver, vertebrae (Fig 4S,T) and skin (Fig 4J,K).

### Expression of human CLCA2 and 4 in the nervous system

**Figure 6:**
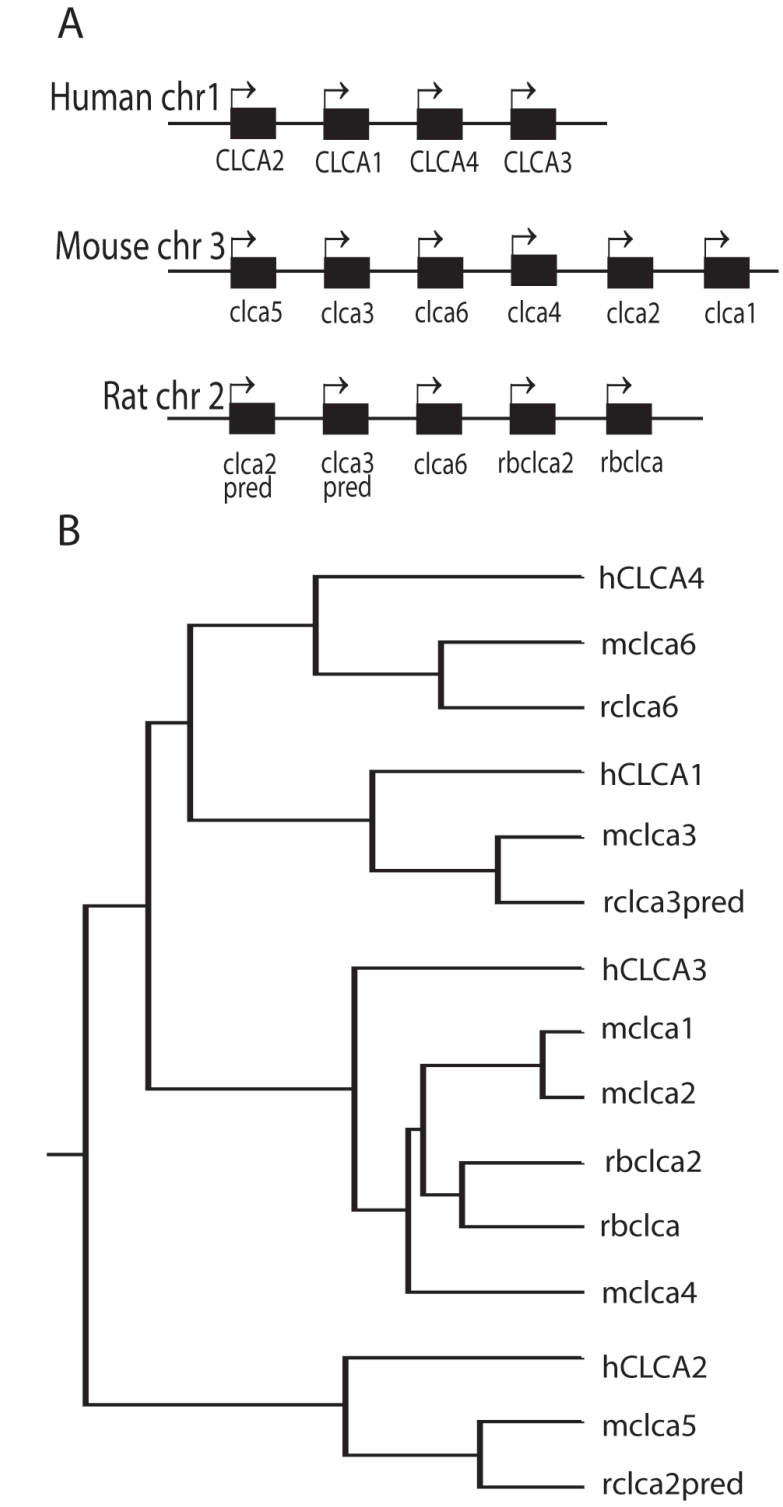
**Structure of human, mouse and rat CLCA locus and homology between CLCA family members**. Schematic depiction of order of succession of genes within human, mouse and rat CLCA locus is shown in A (the figure is not drawn in scale). Homology tree showing relationship between human, mouse and rat CLCA proteins (B). The dendrogram was generated using DNAMAN software (Lynnon Biosoft) with complete amino acid sequences of the CLCA proteins.

Since three mouse Clca gene family members were expressed in brain, we were interested if any of the four human CLCA genes are expressed in the nervous system. As the numbering of CLCA family members in human and rodents is different, we performed bioinformatic analysis to reveal which rodent Clca genes have closest homology to which human family members. Schematic depiction of human, mouse and rat CLCA loci is shown in Fig 6A. We created a homology tree of the mouse, rat and human proteins using DNAMAN software (Fig 6B). Bioinformatic analysis revealed that the order of genes within the rodent and human locus was similar e.g. hCLCA2, which is the most 5' of the human genes has the highest homology with mClca5 and rClca2 (predicted gene), which also lie in the most 5' part of the mouse and rat locus respectively (Fig 6A). Our analysis also showed that the 3' part of the locus has undergone duplication in rat (rbClca and rbClca2 genes) and triplication in mouse (mClca1, 2 and 4 genes), whereas there is a single 3' gene (CLCA3) in the human locus (Fig 6).

**Figure 7:**
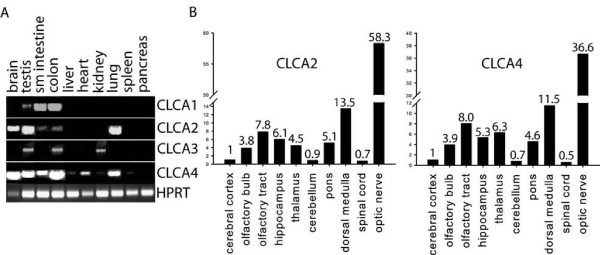
**A) Semiquantitative RT-PCR analysis of human CLCA1-4 mRNA expression and control mRNA hprt in adult human tissues**. B) Real-time PCR analysis of CLCA2 and CLCA4 expression in adult human brain regions. Expression levels are shown relative to the expression level in the cerebral cortex.

Our bioinformatic analysis showed that human CLCA3 is the closest homologue of mouse Clca1, 2 and 4 genes (Fig 6B). It shares 74% homology at the protein level with its mouse counterparts. RT-PCR analysis revealed that neither hCLCA3 nor hCLCA1 was expressed in the nervous system. In contrast, hCLCA2 and hCLCA4 were expressed in the brain (Fig 7A). Expression of hCLCA1 was largely confined to the gastrointestinal tract, with high level of expression in the small intestine. Low level of hCLCA1 expression was detected in testis. hCLCA2 was expressed at high levels in the brain, testis and lung and at low levels in the small intestine and colon. hCLCA3 was expressed in testis, kidney and colon. hCLCA4 was expressed widely in human tissues. High levels of hCLCA4 were expressed in the brain, testis, small intestine, colon and lung. Lower levels of expression were detected in heart (Fig 7A).

hCLCA2 and 4 were differentially expressed in the adult brain as revealed by quantitative RT-PCR analysis (Fig 7B). The highest level of hCLCA2 and hCLCA4 expression was found in optic nerve. The expression level of hCLCA2 and hCLCA4 in optic nerve exceeded 58 and 35 times the expression level of the corresponding gene in cerebral cortex. The expression of hCLCA2 and hCLCA4 was also significantly higher in medulla and olfactory tract as compared to the expression level in cerebral cortex. Very low levels of hCLCA2 and hCLCA4 expression were found in cerebral cortex, cerebellum and spinal cord (Fig 7B).

## Discussion

In this study we show novel expression sites for mouse Clca1, Clca2 and Clca4 genes. All six mouse Clca genes are located in chromosome 3 and are clustered in the same locus. Our RT-PCR and in situ hybridization analyses reveal that in the murine nervous system, mClca1, 2 and 4 genes are preferentially expressed in the olfactory ensheathing cells. In contrast to our findings, it has been previously shown that mClca2 is not expressed in the mouse brain [13]. Our analysis reveals that mClca2 is expressed in mouse brain, albeit at very low levels. The discrepancy between the results may come from the observation that at least in the gastrointestinal tract, there are marked differences in the expression level of mouse Clca genes between different mouse strains [18]. Also, it has recently been shown that mClca2 is expressed in the cerebral cortex, albeit at very low levels as compared to mClca1 [19]. The same study also detected low levels of mClca5 expression in the dorsal root ganglia of adult mouse.

mClca 1, 2 and 4 genes share highest similarity with each other within the gene family. At amino acid level mClca1 and 2 share 95% identity and mClca4 shares 81% identity with mClca2 and 80% identity with mClca1. Moreover, these three genes lie next to each other and form a 3' gene cluster in the Clca gene locus. Given their similar expression pattern in the nervous system, it could be argued that either their olfactory ensheathing cell specific expression is driven by a common regulatory element or each of these mouse genes has retained an olfactory ensheathing cell specific promoter element following gene duplication. Other reports have shown that the rat Clca genes most homologous to mouse Clca1,2 and 4, i.e. rbClca and rbClca2 are expressed in rat brain [20,21]. Their analysis revealed that both genes are expressed at comparable levels in the cerebellum, cerebrum and spinal cord. Also, they showed by single cell PCR analysis that rbClca is expressed in both neurons and glial cells. It would be interesting to analyze the expression level of rbClca and rbClca2 in the olfactory bulb of rat.

Our analysis of mClca1/2 expression in the nervous system at early postnatal development revealed that it is expressed also in the layer II-III of the cerebral cortex. Interestingly the expression was seen only in the frontal part of the developing cortex. At E17 mClca1/2 expression was more widespread in the brain, with prominent expression in diencephalon. During embryonic development mClca1/2 expression was seen in the developing nerves of the peripheral nervous system. We could detect expression at E13 in the developing olfactory nerve and spinal nerves and at E17 in the optic nerve, trigeminal nerve and olfactory nerve. It is possible that mClca1/2 expression marks the glial cells ensheathing peripheral nerves.

In this study we have also analyzed the expression of human CLCA genes in the nervous system. Human CLCA locus contains 4 genes. The most 3' of the genes is hCLCA3, which is also most closely related to mClca1, 2 and 4. RT-PCR analysis showed that unlike its mouse homologues, hCLCA3 is not expressed in the nervous system. In contrast, our results show that two other members of the family, hCLCA 2 and 4 are expressed in various parts of human brain. It has previously been shown, using RNA dot-blot analysis, that hCLCA4 is expressed rather uniformly in the brain with striking absence in the cerebellum [22]. However, RT-PCR analysis performed in this study showed low level of CLCA4 expression in cerebellum. Unlike mouse Clca genes expressed in the nervous system, human CLCA 2 and 4 expression is not confined to olfactory ensheathing cells. It should be noted however, that both genes were expressed at higher levels in the olfactory nerve as compared to olfactory bulb. Highest expression for both genes was found in the optic nerve. Together with the data from the analysis of mouse mClca1/2 expression, it could be suggested that CLCA genes could be expressed at higher levels in the cells that ensheath cranial nerves.

## Conclusion

In this study we have shown that mClca1, mClca2 and mClca4 are expressed in the olfactory ensheathing cells of the adult mouse CNS. During mouse development mClca1/2 widely expressed in the CNS but at particularly high levels in cranial nerves. In addition, we found that mClca1/2 is expressed in layer II of the developing cerebral cortex at P9. Our analysis also reveals that hCLCA2 and hCLCA4 are expressed in the CNS of adult humans.

## Authors' contributions

TT and DM contributed to the design of the experiments and to the preparation of the manuscript. MP performed the experiments and contributed to the design of the experiments and to the preparation of the manuscript.
